# Urethral hemangioma in a prepubertal female patient: Report of a rare case

**DOI:** 10.1097/MD.0000000000006502

**Published:** 2017-03-31

**Authors:** Chiao-Ching Li, Chiao-Zhu Li, Ching-Heng Yen, Wen-Chuan Tsai, Sheng-Tang Wu, Tai-Lung Cha, En Meng

**Affiliations:** a Division of Urology, Department of Surgery; b Department of Neurological Surgery, Tri-Service General Hospital, National Defense Medical Center, Taipei; c Department of Surgery, Kaohsiung Armed Forces General Hospital, Kaohsiung; d Department of Surgery, Tri-Service General Hospital Songshan Branch; e Department of Pathology, Tri-Service General Hospital, National Defense Medical Center, Taipei, Taiwan.

**Keywords:** case report, female, hemangioma, prepubertal, urethra

## Abstract

**Rationale:**

Urethral hemangiomas commonly occur in men or elderly women. We presented a rare case of urethral hemangioma in a prepubertal female patient.

**Patients concerns:**

An 8-year-old girl had the complaints of bloody staining of clothing, a foul perineal odor, and urethral pain. She was brought to our genitourinary outpatient department for survey and a single, 1-cm compressible reddish nodule at the 10-2 o’clock position in the distal urethra was found.

**Diagnoses:**

Urethral hemangioma in a prepubertal girl was diagnosed after excision of the urethral nodule.

**Interventions:**

Excision with 8 “stay” sutures in the protruding urethral mucosa was performed.

**Outcomes:**

Normal micturition without symptom recurrence was noted at the 3-month follow-up.

**Lessons:**

Urethral hemangioma is also found in prepubertal female patient. Increased physician awareness and early recognition of a urethral hemangioma can avoid unnecessary examinations and patient anxiety. The procedure of excision with 8 “stay” sutures in the protruding urethral mucosa facilitates mobilization from the distal urethra and provides a good surgical view of abnormal proliferative blood vessels. Therefore, the lesion can be removed as clean as possible.

## 1 Introduction

Hemangiomas rarely occur in the urethra, and most reported cases have involved men and elderly women. The clinical manifestations of urethral hemangiomas include lower urinary tract symptoms, dysuria, hematuria, perineal discomfort, and urethral discharge. We report a case of urethral hemangioma in an 8-year-old girl, who was brought to our outpatient department because of bloody staining of clothing and a foul perineal odor. Urethral hemangioma was diagnosed after surgery. To the best of our knowledge, this is the youngest reported case ever.

## 2 Case report

**Figure 1:**
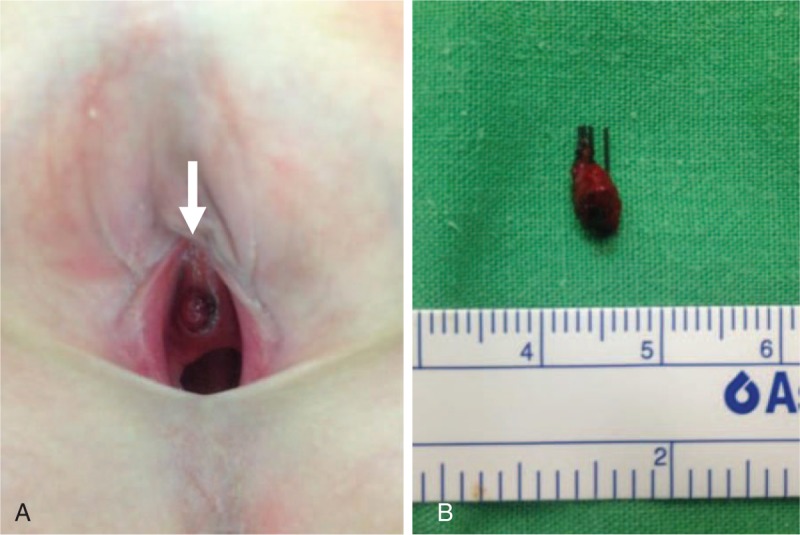
(A) A reddish, soft mass protruding from the distal urethra (arrow). (B) Grossly, the specimen measured 0.8 × 0.5 × 0.3 cm.

A healthy, 8-year-old girl with no hereditary disease was brought to our genitourinary outpatient department because of bloody staining of clothing, a foul perineal odor, and urethral pain. A 1-cm compressible reddish nodule was noted at the 10 to 2 o’clock position in the distal urethra (Fig. 1A). She was admitted for lesion excision. Preoperative laboratory data were normal.

Cystourethroscopy before the operation revealed normal bladder mucosa and bladder neck. An erythematous, protruding mucosal lesion was found in the distal ventral urethra, and the mass was completely excised. The operative method was similar to that used for urethral prolapse. Initially, we performed cystourethroscopy. We placed 8 “stay” sutures in the protruding urethral mucosa to facilitate mobilization from the distal urethra. The transection zone was exposed as a fibrous groove surrounding the distal urethra. We meticulously incised the mucosa to avoid the muscular layer and prevent bleeding, and cautery was used for hemostasis. We carefully excised the protruding lesion held by the stay sutures. Finally, the residual mucosa was attached to the external urethra with 3-0 chromic catgut interrupted sutures.^[1,2]^ Urethral catheterization was performed at the end of the operation.

**Figure 2:**
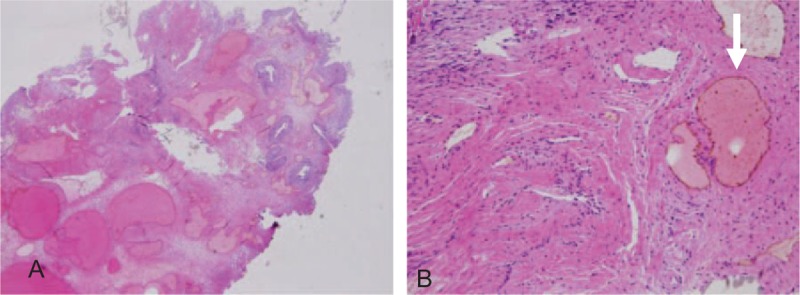
(A) Dilated blood vessels located in the submucosal layer (×40). (B) Small vessel proliferation is also seen in the muscle layer (white arrowheads) (×200).

**Figure 3:**
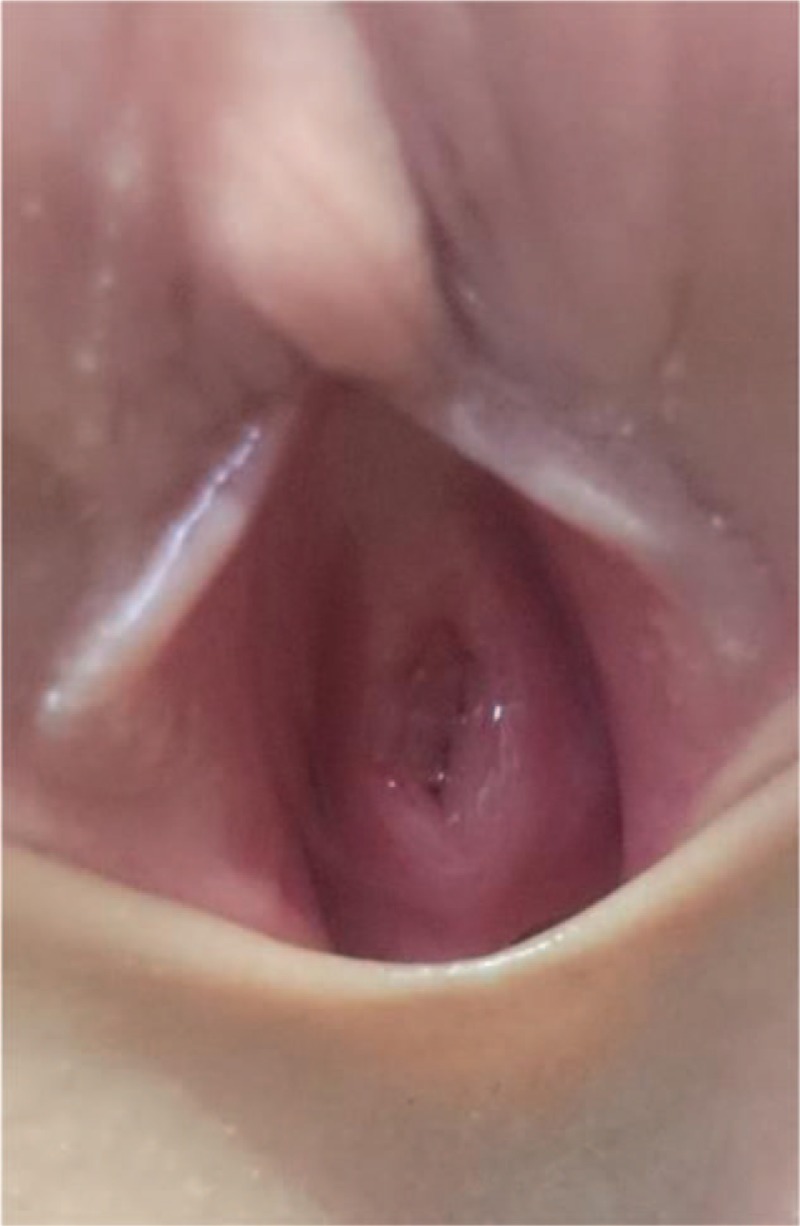
The surgical wound had healed well 1 week after surgery.

Grossly, the specimen measured 0.8 × 0.5 × 0.3 cm. It was red and had a soft texture (Fig. 1B). Microscopically, a few proliferative, thin-walled, and dilated blood vessels were found (Fig. 2A and B). The lesion was compatible with a urethral hemangioma. We removed the urethral catheter on the 2nd postoperative day. The surgical wound had healed well after 1 week (Fig. 3). Normal micturition without symptom recurrence was noted at the 3-month follow-up.

The patient agreed to publication of the case report and her parents gave informed consent. The procedure was approved by the Ethics Committee of the Tri-Service General Hospital.

## 3 Discussion

A hemangioma is an abnormal proliferation of blood vessels that may occur in any vascularized tissue. It is believed to be congenital, arising from embryonic remnants of unipotent angioblastic cells that develop into abnormal blood vessels.^[3]^ Hemangiomas are commonly present in the skin and liver, but are rare in the urinary tract. Urethral hemangiomas are more common in men and are very rarely reported in women.^[3–11]^

The clinical presentations include lower urinary tract symptoms, with dysuria, hematuria, perineal discomfort, and urethral discharge. Physical examination, cystourethroscopy, and 3-dimensional magnetic resonance imaging may help confirm the diagnosis.^[5]^ The differential diagnosis of a circular lesion of the external urethral meatus includes prolapse, polyp, caruncle, periurethral abscess, and malignancy. Urethral prolapse occurs most commonly in prepubertal black and postmenopausal white female patients.^[12–14]^ Our patient was Asian and was not in a high-risk group.

Urethral prolapse is defined as the complete eversion of the terminal urethra from the external meatus. About a quarter of the urethral mucosa protrudes as a caruncle, which consists of connective tissue containing many inflammatory cells and blood vessels, and is often present at the posterior lip of the urethral meatus.^[15,16]^ The protruding nodule in our patient was located in the ventral lip of the urethral meatus, which is not the common location of a caruncle. Common malignancies of the urethra include squamous cell carcinoma, urothelial cell carcinoma, adenocarcinoma, sarcoma, and melanoma.

Treatment of urethral hemangiomas includes observation, oral steroids,^[6]^ excision, and various endoscopic treatments, such as electrocautery or laser ablation.^[5,7]^ It has been reported that 8-week treatment of oral steroids may be used to treat the urethral and vaginal capillary hemangioma in a young child.^[6]^ In our case, the girl had symptoms of bloody staining of clothing and a foul perineal odor. The embarrassed condition bothered her and her family. Although treatment with oral steroids was not invasive, it may not solve the problem immediately and increase the concern of systemic side effects. On the other hand, endoscopic intervention may have a high recurrence rate and is not suitable for a distal urethral lesion. Therefore, we performed wide excision to treat the patient. The procedure provided a good surgical view of abnormal proliferative blood vessels and the lesion can be removed as clean as possible. No recurrence was noted at a 3-month follow-up.

## 4 Conclusion

We presented a case of an 8-year-old girl with a reddish protruding mass in the distal urethra, with occasional bleeding. Wide excision of the urethral mass was performed and the final pathology report was compatible with urethral hemangioma, which is very rare in prepubertal girls. Increased physician awareness and early recognition of a urethral hemangioma avoids unnecessary examinations and patient anxiety. To the best of our knowledge, this is the youngest reported case ever.
